# Spatiotemporal prediction of obesity rates and model interpretability analysis from a public health perspective

**DOI:** 10.1371/journal.pone.0335908

**Published:** 2025-11-13

**Authors:** Weiyan Tan, Bing Geng, XiuGuang Bai

**Affiliations:** Guangdong Service Center for Veterans, Guangzhou, Guangdong, China; National University of Defense Technology, CHINA

## Abstract

This study, focusing on the assessment of obesity prevalence trends in public health management, proposes an improved Transformer model that integrates temporal embeddings with spatially-constrained feature dependencies rather than purely geographic adjacency. Using state-level data from the CDC BRFSS, the method first performs joint temporal–health encoding (JTH) of obesity prevalence time series and health indicators. It then incorporates temporal decay and a learnable spatial constraint matrix (STA) into the attention mechanism, while employing dual-branch consistency training to enhance stability and generalization. We conducted comparative and ablation experiments on ten states, including Alaska and Alabama, and carried out independent validation on unseen states such as Guam and Idaho. The results show that the proposed approach outperforms representative models including MLP, LSTM, 1D-CNN, Mamba, iTransformer, and TimeMixer across metrics such as MAE, RMSE, sMAPE, R^2^, and MASE. Ablation experiments further demonstrate that JTH and STA contribute complementary improvements to model performance, while independent validation confirmed that the R^2^ values for all states exceeded 0.84. In addition, SHAP analysis was employed to illustrate the contributions and dependencies of key features, providing interpretable evidence to support, thereby guiding evidence-based resource allocation in obesity prevention and control.

## 1 Introduction

In recent years, public health issues have received increasing global attention, with the high prevalence of obesity emerging as a major challenge threatening human health and social development [1,2]. Reports from the World Health Organization indicate that obesity is not only highly associated with cardiovascular disease, diabetes, and various cancers, but also places long-term pressure on public health systems [3–5]. Therefore, accurately predicting the dynamic trends of obesity prevalence and uncovering its potential driving factors are of great importance for designing targeted interventions and public policies. In regions with significant socioeconomic disparities and complex environmental factors, spatiotemporal modeling and interpretability analysis provide new data-driven approaches for public health decision-making [6]. Table 1 shows the obesity rate trends in different regions.

**Table 1:** Overview of obesity prevalence and trend studies across different regions.

Study Scope	Time Period	Study Population	Main Findings
Global (191 countries) [7]	1990–2022	Children, adolescents, adults	Obesity continued rising; in 2022 about 14% of men and 18.5% of women were obese; notable regional disparities.
Global (200+ countries) [8]	1975–2016	Children, adolescents, adults	BMI increased steadily; childhood and adolescent obesity rapidly approached adult levels.
Global (195 countries) [9]	1990–2015	Adults, children	Burden from overweight/obesity rose substantially; Middle Eastern and Pacific Island nations among the highest rates.
United States (national & state) [10]	1990–2021; projected to 2050	Children, adolescents, adults	In 2021, U.S. adult obesity exceeded 40%; most states are projected to surpass 50% by 2050.
China (urban & rural) [11]	2004–2018	Adults	Adult BMI and obesity rose rapidly; urban–rural gaps narrowed; increases especially pronounced among women.

However, existing studies still face numerous challenges in modeling and applying public health data. First, traditional statistical methods often struggle to effectively capture the nonlinear spatiotemporal evolution of obesity prevalence, resulting in limited predictive accuracy. Second, while certain deep learning methods can improve prediction performance to some extent, they often lack sufficient interpretability, making it difficult to answer the question of “which factors are most critical to obesity changes across different regions and time periods.” Furthermore, public health data typically exhibit multi-source heterogeneity, missing values, and imbalances, further complicating model generalization and real-world applicability. These challenges limit the credibility and practical value of predictive models in public health decision-making.

To address these issues, this paper proposes a predictive framework that integrates temporal modeling with spatial constraints, while explicitly incorporating interpretability mechanisms into the model architecture. Unlike prior interpretable spatiotemporal deep learning approaches such as GNN-based models or hybrid RNN–Transformer variants, our method introduces a unified design that combines temporal embeddings, feature-level spatial constraints, and consistency training. Specifically, we design a modeling module based on temporal embeddings and joint encoding of health indicators to enhance the ability to capture complex dynamic patterns. At the same time, by embedding feature-constrained spatial relations rather than purely geographic adjacency into the attention mechanism, the model can better characterize spatiotemporal dependencies among regions. In the prediction stage, we adopt a multi-branch consistency training strategy to improve model stability and generalization, and employ SHAP-based methods to achieve feature-level interpretability analysis. Through this integrated approach, the framework provides both accurate forecasting of obesity prevalence and transparent, interpretable evidence to support public health decision-making.

The main contributions of this paper are summarized as follows:

(1) From a public health perspective, we systematically propose a spatiotemporal modeling framework for obesity prediction that effectively integrates temporal embeddings and spatial adjacency relationships;

(2) We incorporate interpretability mechanisms into the model architecture, leveraging feature importance analysis and visualization to identify the key driving factors of obesity prevalence;

(3) We develop a multi-branch consistency training strategy that enhances model stability and generalization in cross-regional and cross-temporal predictions;

(4) We conduct extensive experiments on multiple public health datasets, demonstrating that the proposed method outperforms existing baselines in both predictive accuracy and interpretability, thus providing practical references for public health interventions and policymaking.

## 2 Related work

### 2.1 Spatiotemporal modeling and public health data analysis

The importance of spatiotemporal modeling in public health research has become increasingly prominent. A growing body of literature has employed spatial statistical models, time series methods, and deep learning approaches to analyze the spatiotemporal dynamics of obesity and related chronic diseases. Gao *et al*. [12] modeled and projected childhood obesity trends in 191 countries from 1975 to 2030, revealing long-term cross-national patterns. Guo *et al*. [13] utilized multiple waves of Chinese nutrition and health survey data, applying Bayesian models with environmental features to predict childhood and adolescent obesity. Similarly, Tong *et al*. [14] used data from 2016–2020 on Chinese adolescents and found clear regional clustering patterns of obesity, while Azanaw *et al*. [15] demonstrated that socioeconomic factors significantly explained spatiotemporal distributional differences across years in their study of Ethiopian urban women. Collectively, these studies highlight the importance of incorporating spatial and temporal dimensions, providing a solid empirical foundation for understanding the dynamic characteristics of obesity prevalence.

In terms of methodological development, scholars have sought to integrate predictive models with multi-source data to enhance the interpretability and predictive capacity of public health research. For example, Shiri *et al*. [16] employed a Bayesian spatiotemporal model to forecast obesity prevalence in Iran through 2040, uncovering long-term effects of gender differences and regional characteristics. Grimaccia and Rota [17] analyzed the spatiotemporal dynamics of obesity in Italian regions and stressed the need for regional interventions. At the same time, advanced modeling techniques have been gradually introduced into public health contexts. The EpiGNN model [18], leveraging graph neural networks, revealed regional transmission mechanisms and provided insights into spatial dependence predictions for chronic diseases. Çolak [19] applied multiple time series models to predict the long-term obesity trends among older adults in the United States, whereas Dahu *et al*. [20] combined satellite remote sensing imagery with deep convolutional networks to achieve high-precision predictions of obesity prevalence in Missouri. Furthermore, Rota *et al*. [21] proposed a spatiotemporal dynamic modeling framework based on Bayesian Beta regression, offering a novel tool to explore regional differences and future trajectories of obesity prevalence. These studies not only demonstrate the application potential of diverse methodologies in public health but also provide feasible pathways for prediction and decision support in the prevention and control of obesity and other chronic diseases.

### 2.2 Model interpretability and health decision support

In public health research, model interpretability plays a critical role in ensuring the scientific validity of results and their value for decision support. Recent studies have widely applied explainable artificial intelligence (XAI) methods to uncover the underlying factors in obesity prediction models. Allen [22] developed an interpretable machine learning model for county-level obesity rates in the United States, demonstrating the contributions of socioeconomic and environmental variables. Görmez *et al*. [23] combined lifestyle and dietary habit data with ensemble XAI-based machine learning methods to achieve transparent prediction of obesity levels. Du *et al*. [24] designed a visualization-based risk prediction system, translating model outputs into intuitive health management tools. Khater *et al*. [25] further revealed the complex mechanisms of lifestyle factors influencing obesity. Lin *et al*. [26] proposed an interpretable obesity risk prediction model for overweight populations, providing valuable references for individual interventions. Meanwhile, scholars have also explored general interpretable classification models [27], as well as explainable prediction approaches based on electronic health records (EHR) [28], underscoring the close connection between data-driven methods and clinical applications.

In the context of health decision support, interpretability not only enhances model transparency but also strengthens its practical utility in clinical and policy settings. Cho *et al*. [29] applied interpretable models to predict postoperative hospital stay, verifying their feasibility in clinical resource management. Amarasinghe *et al*. [30] emphasized the potential and research gaps of explainable machine learning in public policy applications. Gupta *et al*. [31] proposed an obesity prediction framework that integrates deep learning with interpretable elements based on EHR data, while Deva *et al*. [32] highlighted the limitations of “non-identifiability” in constraining model interpretability from an epidemiological perspective. Kumar *et al*. [33] introduced concept-driven self-explainable neural networks for ICU mortality prediction, demonstrating that it is possible to balance high performance with interpretability in complex medical scenarios. Overall, these studies underscore the critical role of XAI technologies in health prediction, resource allocation, and policy-making, providing more reliable scientific evidence for obesity prevention and public health management.

## 3 Method

### 3.1 Ethics statement

This study only uses state-level aggregated data (BRFSS dataset) publicly released by the U.S. Centers for Disease Control and Prevention (CDC), and does not involve any identifiable personal information or individual privacy. Therefore, this study does not require additional ethical approval.

### 3.2 Overall model architecture

**Fig 1:**
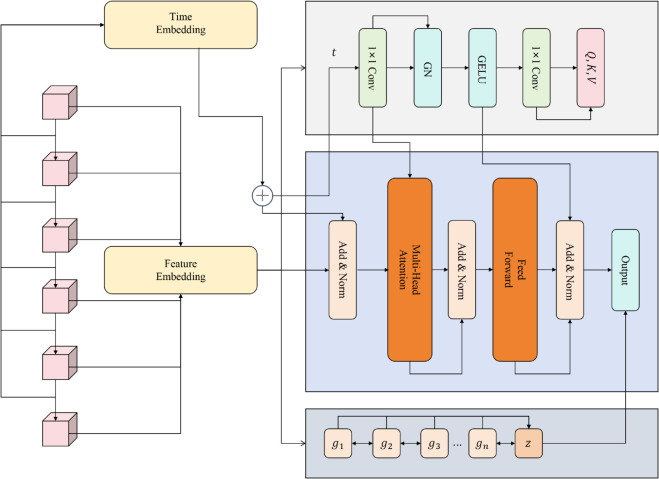
Overall model architecture. The model takes temporal embeddings and indicator embeddings as inputs, and leverages the improved spatiotemporal constrained attention module to capture long-term dependencies and dynamic variations. The final output layer not only maintains predictive accuracy but also enhances interpretability, providing effective support for obesity rate prediction in public health scenarios.

This study proposes a model based on an improved Transformer architecture for spatiotemporal prediction of obesity rates within a single state. The overall framework takes time series data as the core input, where positional encoding and feature embedding of health survey indicators are jointly modeled to effectively capture dynamic changes across different years and the associations among public health factors. On this basis, spatiotemporal constraints are incorporated into the attention mechanism, enabling the model to more precisely focus on key features driving the temporal evolution of obesity rates, thereby enhancing both predictive accuracy and interpretability. The design highlights two main innovations: first, the integration of temporal embeddings with health indicators strengthens the model’s ability to represent long-term trends; second, the modified attention mechanism improves spatiotemporal dependency modeling and provides greater interpretability by uncovering the critical driving factors behind obesity rate variations. The overall model architecture is shown in Fig 1.

### 3.3 Joint modeling of time series embedding and health indicators (JTH)

In the overall modeling process, we treat the public health data sequences related to obesity rates as time series signals, denoted as $\{y_{t}{\}}_{t=1}^{T}$. To capture their temporal dynamics more concisely, we define an embedding function that projects each raw input into a *d*-dimensional latent space:

$$h_{t}=f_{\text{embed}}(y_{t},t),$$ (1)

where $h_{t}\in {\mathbb{R}}^{d}$ is the representation at time *t*. This embedding allows the model to represent long-term trends and short-term fluctuations within a unified feature space. In this study, $f_{\text{embed}}$ combines the raw input value with a sinusoidal positional encoding of the time index *t*, rather than a learnable embedding. The sinusoidal encoding enables the model to capture periodicity and relative temporal information effectively, while ensuring that embeddings for unseen time steps can be consistently extrapolated.

**Fig 2:**
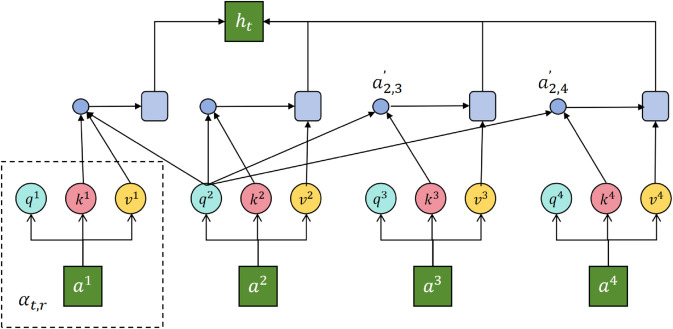
Illustration of the attention mechanism in the joint modeling module. This component constructs interactive attention between time series embeddings and health indicators, effectively capturing dependencies across different time segments, thereby enhancing the model’s representational capacity and interpretability in obesity rate prediction.

On this basis, we introduce a multi-head attention mechanism to jointly model the embeddings and obtain global dependencies across time steps. Specifically, let $Q_{t}=W_{Q}h_{t}$, $K_{t}=W_{K}h_{t}$, and $V_{t}=W_{V}h_{t}$, then for any time step *t*, its dependency on other time steps can be expressed as

$$\alpha_{t,\tau }=\frac{\exp\left(Q_{t}\cdot K_{\tau }^{⊤}/\sqrt{d}\right)}{\sum_{j=1}^{T}\exp\left(Q_{t}\cdot K_{j}^{⊤}/\sqrt{d}\right)},$$ (2)

$$z_{t}=\sum_{\tau =1}^{T}\alpha_{t,\tau }V_{\tau },$$ (3)

where $\alpha_{t,\tau }$ denotes the attention weight and *z*_*t*_ is the aggregated context representation. In this way, the model can explicitly emphasize the most critical time segments for prediction, thereby avoiding the gradient vanishing problem when modeling long-term dependencies. The attention mechanism architecture is shown in Fig 2.

**Fig 3:**
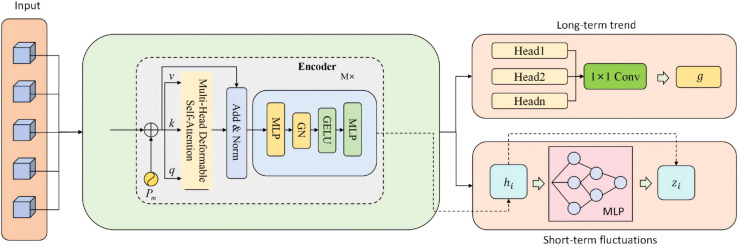
Joint modeling of time series embedding and health indicators. The framework captures long-term trends via convolution-based aggregation and short-term fluctuations via MLP-based encoding, providing complementary temporal representations.

To further stabilize the prediction results and enhance interpretability, we introduce a temporal residual connection after context modeling, namely

$${\tilde{h}}_{t}=h_{t}+z_{t},$$ (4)

which ensures that the original temporal information is not lost during deep propagation, effectively preserving sensitivity to short-term dynamics. Meanwhile, the residual term provides an additional channel for interpretability analysis, enabling us to distinguish the respective contributions of the original trend and the global dependencies in the prediction process. The model architecture is shown in Fig 3.

Finally, the joint representations of all time steps are aggregated to obtain the overall prediction vector. The specific form is

$$g=\text{Aggregate}\left(\{{\tilde{h}}_{t}{\}}_{t=1}^{T}\right),$$ (5)

$${\hat{y}}_{T+1}=f_{\text{pred}}(g),$$ (6)

where ${\hat{y}}_{T+1}$ denotes the predicted obesity rate at the next time step, and $f_{\text{pred}}$ is the prediction mapping function. Through the joint modeling of time series embeddings and health indicators, the model can simultaneously capture long-term trends and critical changes, ensuring stable predictive performance while enhancing interpretability, thereby making it highly valuable for public health research.

### 3.4 Attention mechanism with spatiotemporal constraints (STA)

**Fig 4:**
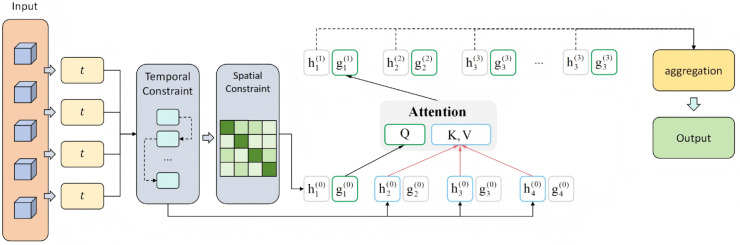
Attention mechanism with spatiotemporal constraint. Temporal decay and correlation-based spatial weights are integrated into the attention computation, enabling the model to capture both local temporal dependencies and cross-feature correlations for improved prediction.

On the basis of joint modeling, to further improve prediction accuracy and stability, we introduce a spatiotemporal constraint mechanism into the attention computation. Let the time series length be *T* and the set of spatial locations be 𝒮, then the representation for any time step *t* and location s∈𝒮 can be written as

$$u_{t,s}\in {\mathbb{R}}^{d},$$ (7)

where *u*_*t*,*s*_ denotes the embedding representation of location *s* at time *t*. In this study, the notion of "spatial" does not correspond to explicit geographic or inter-state adjacency. Instead, it refers to feature-level positions within the embedding space, where spatiotemporal constraints are imposed to regulate correlations among different feature dimensions across time. In other words, the spatial dimension here is an abstraction of learned feature interactions, rather than a physical or population-based neighborhood structure. This setting enables the model not only to capture dependencies along the temporal dimension, but also to model potential correlations across spatial positions. The model architecture is shown in Fig 4.

To encode spatiotemporal constraints within the attention mechanism, we first define a time-dependent decay function:

$$\omega_{t,\tau }=\exp\left(-\frac{\left|t-\tau \right|}{\lambda_{t}}\right),$$ (8)

where $\lambda_{t}$ is a temporal scale hyperparameter that controls the decay rate between different time steps. This mechanism ensures that the model remains sensitive to neighboring time points while modeling long-term dependencies.

Similarly, in the spatial dimension we introduce a correlation-based matrix $A\in {\mathbb{R}}^{\left|𝒮|\times |𝒮\right|}$ and define the spatial constraint function as

$$\phi_{s,r}=\frac{A_{s,r}}{\sum_{j\in 𝒮}A_{s,j}},$$ (9)

where *A*_*s*,*r*_ is computed from the correlation coefficient between feature position *s* and feature position *r* in the embedding space, rather than from geographic adjacency. This correlation-based spatial constraint guides the attention mechanism to focus aggregation on feature dimensions that exhibit stronger statistical associations or contextual relevance.

Based on the above definitions, the spatiotemporally constrained attention weights can be expressed as

$$\beta_{\left(t,s),(\tau ,r\right)}=\frac{\exp\left(\psi (u_{t,s},u_{\tau ,r})\cdot \omega_{t,\tau }\cdot \phi_{s,r}\right)}{\sum_{\tau =1}^{T}\sum_{r\in 𝒮}\exp\left(\psi (u_{t,s},u_{\tau ,r})\cdot \omega_{t,\tau }\cdot \phi_{s,r}\right)},$$ (10)

where ψ(·,·) denotes a similarity function. This formulation explicitly integrates temporal decay and spatial adjacency into the attention computation, enabling the model to simultaneously focus on critical time segments and spatial locations during inference.

After obtaining the constrained attention weights, we aggregate the context representations as

$$v_{t,s}=\sum_{\tau =1}^{T}\sum_{r\in 𝒮}\beta_{\left(t,s),(\tau ,r\right)}\;u_{\tau ,r}.$$ (11)

Furthermore, to enhance the model’s ability to capture global dynamics, we perform weighted aggregation of the temporal context vectors across all spatial positions:

$$g_{t}=\sum_{s\in 𝒮}\eta_{s}\;v_{t,s},$$ (12)

where $\eta_{s}$ denotes a learnable spatial weight coefficient.

Finally, the overall representation of the sequence is obtained through temporal aggregation:

$$z=\text{Aggregate}\left(\{g_{t}{\}}_{t=1}^{T}\right),$$ (13)

and the final result is produced by the prediction function:

$${\hat{y}}_{T+1}=f_{\text{out}}(z).$$ (14)

By incorporating spatiotemporal constraints into the attention mechanism, the model not only captures long-term dependencies while avoiding over-smoothing, but also maintains sensitivity to critical time segments. At the same time, the spatial constraint ensures that the prediction results align more closely with the propagation logic observed in real-world public health processes. Overall, this design improves predictive accuracy while also revealing the spatiotemporal driving factors behind obesity rate variations, thereby enhancing interpretability.

**Algorithm 1. Prediction process of spatiotemporal constrained attention.**



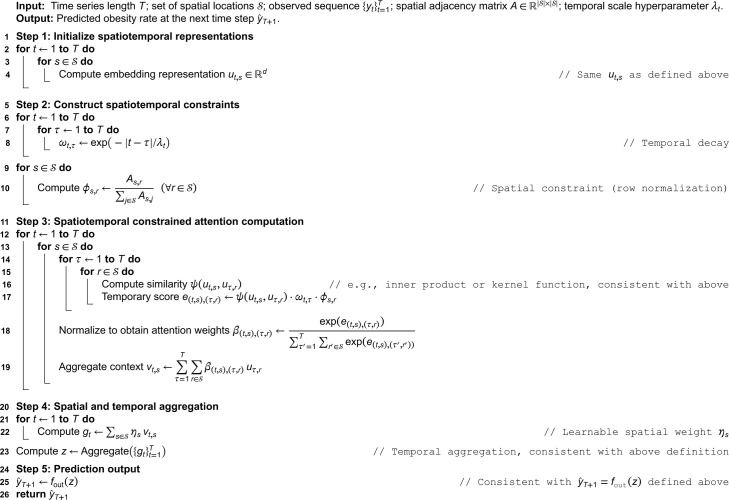



### 3.5 Training objectives

During the training phase, we jointly consider the two outputs obtained from the time series embedding and the spatiotemporal attention constraint. Let $g=\text{Aggregate}(\{{\tilde{h}}_{t}{\}}_{t=1}^{T})$, and obtain the prediction result through $f_{\text{pred}}(g)$; meanwhile, let $z=\text{Aggregate}(\{g_{t}{\}}_{t=1}^{T})$, and obtain another prediction value through $f_{\text{out}}(z)$. Here, $f_{\text{pred}}(g)$ is regarded as the temporal branch, focusing on sequential embeddings, while $f_{\text{out}}(z)$ serves as the spatiotemporal branch, emphasizing correlations constrained by attention. Both branches are trained simultaneously, and their outputs are optimized against the ground-truth label *y*_*T* + 1_.

Specifically, we adopt the Mean Squared Error (MSE) as the optimization objective, defined as

$${ℒ}_{\text{MSE}}=\frac{1}{N}\sum_{i=1}^{N}{\left(y_{T+1}^{\left(i\right)}-{\hat{y}}_{T+1}^{\left(i\right)}\right)}^{2},$$ (15)

where ${\hat{y}}_{T+1}^{\left(i\right)}$ jointly comes from $f_{\text{pred}}(g)$ and $f_{\text{out}}(z)$. The final objective function can be expressed as

$$ℒ=\alpha \cdot {ℒ}_{\text{MSE}}^{\text{pred}}+\beta \cdot {ℒ}_{\text{MSE}}^{\text{out}},$$ (16)

where ${ℒ}_{\text{MSE}}^{\text{pred}}$ and ${ℒ}_{\text{MSE}}^{\text{out}}$ correspond to the temporal and spatiotemporal branches, respectively, and α,β are weighting coefficients that control their relative contributions. In this study, we set α=β=1 to balance the two paths. During inference, the final prediction ${\hat{y}}_{T+1}$ is obtained as the weighted average of the two branches: ${\hat{y}}_{T+1}=\frac{1}{2}(f_{\text{pred}}(g)\;+\;f_{\text{out}}(z))$. This design ensures that both the temporal and spatiotemporal perspectives contribute to the final result, thereby improving stability and robustness.

## 4 Dataset and evaluation metrics

### 4.1 Dataset

This study uses data from the Behavioral Risk Factor Surveillance System (BRFSS) of the Centers for Disease Control and Prevention (CDC), which covers public health information related to adult diet, physical activity, and weight status. The data are included in the Data, Trends, and Maps database of the Division of Nutrition, Physical Activity, and Obesity (DNPAO), providing trends on obesity, nutrition, physical activity, and breastfeeding at both the national and state levels. In this study, we selected data from ten states for analysis, namely Alaska (AK), Alabama (AL), Arkansas (AR), Arizona (AZ), California (CA), Colorado (CO), Connecticut (CT), District of Columbia (DC), Delaware (DE), and Florida (FL). Table 2 provides a detailed description of the dataset features. In terms of data preprocessing, we first examined missing values and found that each state contained approximately 10% missing entries, which were randomly distributed. To ensure data integrity, we applied mean imputation for continuous variables and mode imputation for categorical ones. Outliers were detected using the interquartile range method and subsequently replaced with the mean value of the corresponding feature. Finally, all features were normalized using z-score standardization to improve training stability.

**Table 2:** Main columns and descriptions.

Column Name	Description
YearStart / YearEnd	Range of data years
LocationAbbr / LocationDesc	State abbreviation and name
Class / Topic / Question	Survey theme and question description
Data_Value	Corresponding measurement value (obesity rate)
Data_Value_Unit	Measurement unit (percentage)
Data_Value_Type	Type of data value (raw or weighted)
Sample_Size	Sample size information
GeoLocation	Geographic location information
Stratification1	Stratification variable (gender, age group)

In terms of feature engineering, we selected Data_Value (obesity rate) as the primary prediction target, while YearStart, Sample_Size, and Stratification1 were retained as auxiliary explanatory variables. To capture temporal dynamics, we further constructed lag features (one- and two-year lags), first-order and second-order differences, and rolling means with windows of three and five years. These derived features were designed to represent short-term fluctuations, long-term trends, and seasonal effects in obesity rates. All categorical variables such as Stratification1 were converted into one-hot encodings to ensure compatibility with the model. We chose this feature set because it balances interpretability with predictive power, and preliminary correlation analysis confirmed that these engineered variables had significant associations with the target variable.

In addition, we summarize the number of test samples for each state in Table 3. Reporting the sample size ensures transparency of the evaluation process, as the reliability of statistical tests and confidence intervals can be influenced by the scale of the test data. The variation in the number of samples across states also highlights potential heterogeneity in data availability, which may affect model robustness.

**Table 3:** Number of test samples across ten states.

State	Test Samples
Alaska (AK)	252
Alabama (AL)	218
Arkansas (AR)	239
Arizona (AZ)	146
California (CA)	177
Colorado (CO)	169
Connecticut (CT)	198
District of Columbia (DC)	270
Delaware (DE)	262
Florida (FL)	105

### 4.2 Evaluation metric

In the evaluation of model performance, we selected five commonly used metrics to measure the accuracy and stability of the predictions. The specific definitions are as follows.

Mean Absolute Error (MAE) measures the average absolute difference between predicted values and true values. A smaller value indicates higher prediction accuracy:

$$\text{MAE}=\frac{1}{N}\sum_{i=1}^{N}\left|y_{i}-{\hat{y}}_{i}\right|.$$ (17)

Root Mean Squared Error (RMSE) is more sensitive to larger errors and reflects the overall level of prediction bias:

$$\text{RMSE}=\sqrt{\frac{1}{N}\sum_{i=1}^{N}{\left(y_{i}-{\hat{y}}_{i}\right)}^{2}}.$$ (18)

Symmetric Mean Absolute Percentage Error (sMAPE) is a relative error measure that eliminates the effect of different scales, defined as:

$$\text{sMAPE}=\frac{100\%}{N}\sum_{i=1}^{N}\frac{\left|y_{i}-{\hat{y}}_{i}\right|}{\left(|y_{i}|+|{\hat{y}}_{i}|\right)/2}.$$ (19)

The Coefficient of Determination (*R*^2^) measures the proportion of variance in the true values explained by the model. Its range is (−∞,1], and values closer to 1 indicate better fitting performance:

$$R^{2}=1-\frac{\sum_{i=1}^{N}{\left(y_{i}-{\hat{y}}_{i}\right)}^{2}}{\sum_{i=1}^{N}{\left(y_{i}-\bar{y}\right)}^{2}}.$$ (20)

Mean Absolute Scaled Error (MASE) normalizes the error by comparing it with the error of the naive forecasting method, facilitating comparisons across different time series:

$$\text{MASE}=\frac{\frac{1}{N}\sum_{i=1}^{N}\left|y_{i}-{\hat{y}}_{i}\right|}{\frac{1}{N-1}\sum_{i=2}^{N}\left|y_{i}-y_{i-1}\right|}.$$ (21)

## 5 Experimental results and analysis

### 5.1 Experimental setup

In the experimental process, we modeled and predicted obesity rate data from ten states based on the aforementioned improved spatiotemporal attention model. The model input consisted of time series data mapped through embedding, which were jointly modeled using an attention mechanism combining temporal embeddings and spatial constraints, and finally processed by a prediction function to output the estimated obesity rates. During training, we consistently adopted Mean Squared Error (MSE) as the optimization objective, and applied state-specific independent modeling to ensure that the experimental results could faithfully reflect the dynamic characteristics of different regions.

To guarantee reproducibility and fairness of the experiments, we maintained consistent hyperparameter settings across all experiments. Specifically, the model was trained with a fixed learning rate and batch size, while the number of iterations was controlled within a reasonable range to avoid overfitting. In addition, appropriate hidden dimensions were set for both the attention mechanism and the feed-forward network. More concretely, the overall architecture consisted of two stacked spatiotemporal attention layers, each followed by a feed-forward sub-layer. The multi-head attention module used 8 heads, with each head operating on a sub-dimension of 16 (total embedding dimension 128). The feed-forward network contained two fully connected layers with dimensions 256 and 128, respectively. We applied the ReLU activation function after each feed-forward layer, and a dropout rate of 0.1 was used to improve generalization. Layer normalization was applied after both the attention and feed-forward sub-layers. The main hyperparameter configurations are presented in Table 4.

**Table 4:** Main hyperparameter settings.

Hyperparameter	Value
Learning rate	0.001
Batch size	64
Epochs	100
Embedding dimension	128
Attention heads	8 (16 dimensions per head)
Number of attention layers	2
Feed-forward dimensions	256 → 128
Activation function	ReLU
Dropout rate	0.1
Optimizer	Adam
Loss function	MSE

### 5.2 Comparative experimental results

To validate the effectiveness of the proposed method, this study selected a variety of representative time series forecasting models for comparative experiments. The baseline models include traditional neural network approaches (MLP [34], LSTM [35], 1D-CNN [36], BiLSTM [37]), as well as several recently proposed advanced architectures (iTransformer [38], TimeMixer [39], Mamba [40], and LSTM-Transformer [41]). By comparing the performance of different models under the same data and task settings, we can more comprehensively evaluate the advantages and applicability of the proposed method in obesity rate prediction. The experimental results of the average evaluation metrics are shown in Table 5. And The complete state-wise experimental results are provided in the Appendix of Table 12.

**Table 5:** Comparison of average experimental results across ten states (mean ± standard deviation). This is the average result of three random seeds, and all subsequent tables are in this way.

Method	MAE	RMSE	sMAPE	*R* ^*2*^	MASE
MLP	2.561 ± 0.073	4.110 ± 0.102	8.627 ± 0.154	0.883 ± 0.006	0.420 ± 0.012
LSTM	2.213 ± 0.065	3.568 ± 0.091	7.732 ± 0.138	0.902 ± 0.005	0.362 ± 0.010
BiLSTM	2.048 ± 0.059	3.335 ± 0.087	7.379 ± 0.125	0.912 ± 0.005	0.334 ± 0.009
1D-CNN	1.929 ± 0.054	3.143 ± 0.082	6.959 ± 0.118	0.919 ± 0.004	0.311 ± 0.008
Mamba	1.797 ± 0.050	2.912 ± 0.078	6.484 ± 0.111	0.928 ± 0.004	0.284 ± 0.007
LSTM-Transformer	1.676 ± 0.047	2.744 ± 0.073	6.090 ± 0.106	0.925 ± 0.004	0.260 ± 0.007
iTransformer	1.583 ± 0.043	2.594 ± 0.069	5.771 ± 0.099	0.941 ± 0.003	0.243 ± 0.006
TimeMixer	1.581 ± 0.042	2.489 ± 0.067	5.316 ± 0.094	0.943 ± 0.003	0.224 ± 0.006
Ours	**1.502 ± 0.040**	**2.461 ± 0.066**	**4.615 ± 0.089**	**0.949 ± 0.003**	**0.202 ± 0.005**

From the overall comparison, it can be observed that traditional neural network methods such as MLP, LSTM, and BiLSTM are able to capture certain temporal dependencies in obesity rate prediction, but they still suffer from insufficient accuracy when dealing with complex temporal dynamics. Models based on convolution or improved architectures (e.g., 1D-CNN, Mamba, LSTM-Transformer) achieve further improvements in error control and stability, indicating that deeper modeling of time series is of significant value for forecasting public health data. Meanwhile, recently proposed architectures such as iTransformer and TimeMixer outperform traditional methods on most metrics, demonstrating the potential of novel temporal architectures in health trend prediction.

Among all methods, the proposed improved spatiotemporal attention model achieves the best performance, obtaining the lowest values in MAE, RMSE, sMAPE, and MASE, while reaching the highest score in *R*^2^. These results indicate that the proposed method not only provides more accurate predictions of obesity rate trends but also offers more reliable evidence for public health interventions. In addition, when analyzing the results across different states, we observe that the predictive performance is not uniformly distributed. For example, states with relatively stable obesity trends exhibit lower variance in prediction errors, whereas states with stronger fluctuations or more irregular patterns show larger deviations. This suggests that regional characteristics, including population structure, lifestyle, and survey sample size, contribute to heterogeneous prediction difficulty. By explicitly comparing these state-level differences, our model demonstrates robustness in both stable and volatile regions, further supporting its generalization ability.

Beyond numerical superiority, we further analyze the reasons behind the performance gains. Compared to LSTM and BiLSTM, our method better captures long-term dependencies through the temporal embedding mechanism and multi-head attention, avoiding gradient decay issues inherent in recurrent models. Relative to iTransformer, the introduction of the Spatiotemporal Attention module allows our model to incorporate cross-feature relational constraints, which is particularly beneficial for capturing interactions among lag, difference, and rolling mean features that iTransformer tends to treat independently. In comparison with TimeMixer, our Joint Temporal-Health encoding explicitly integrates temporal signals with domain-specific health indicators, thereby strengthening the alignment between feature dynamics and health outcomes. These design choices collectively explain why the proposed model not only improves error metrics but also provides stronger interpretability and adaptability across diverse state contexts.

In real-world scenarios, this means that relevant authorities can identify potential risk distributions of obesity earlier and more precisely, thereby formulating more scientific nutrition interventions and physical activity promotion policies, ultimately providing strong support for public health management and disease prevention. The experimental results of the significance test (paired t-test) are further given, as shown in Table 6.

**Table 6:** Statistical significance tests (p-values) of Ours vs. baselines across five metrics. Results marked with * indicate *p* < 0.05.

Baseline	MAE	RMSE	sMAPE	R^2^	MASE
MLP	$1.29\times 10^{-7}$	$4.21\times 10^{-8}$	$2.25\times 10^{-8}$	$3.37\times 10^{-8}$	$6.27\times 10^{-9}$
LSTM	$3.12\times 10^{-6}$	$7.84\times 10^{-6}$	$2.95\times 10^{-6}$	$1.02\times 10^{-5}$	$5.43\times 10^{-6}$
BiLSTM	$1.77\times 10^{-6}$	$1.17\times 10^{-5}$	$5.77\times 10^{-7}$	$8.76\times 10^{-6}$	$4.57\times 10^{-8}$
1D-CNN	$4.66\times 10^{-6}$	$2.31\times 10^{-4}$	$1.06\times 10^{-6}$	$5.41\times 10^{-5}$	$4.85\times 10^{-8}$
Mamba	$1.57\times 10^{-5}$	$1.95\times 10^{-3}$	$1.06\times 10^{-5}$	$2.20\times 10^{-4}$	$2.66\times 10^{-6}$
iTransformer	$2.69\times 10^{-3}$	$2.02\times 10^{-1}$	$1.94\times 10^{-4}$	$1.04\times 10^{-2}$	$1.63\times 10^{-4}$
TimeMixer	$9.04\times 10^{-3}$	$7.72\times 10^{-1}$	$2.82\times 10^{-3}$	$6.62\times 10^{-2}$	$6.64\times 10^{-4}$

In the results of the statistical significance tests, it can be observed that our method demonstrates significant advantages over most baseline models in key metrics such as MAE, sMAPE, and MASE (*p*<0.05), indicating that the performance improvement is not due to random fluctuations but represents stable and reliable gains. At the same time, for certain metrics such as RMSE and R^2^, the differences compared with the strongest baseline (e.g., TimeMixer) do not reach the level of statistical significance, suggesting that there is still room for further optimization in specific scenarios. Overall, the significance analysis verifies the effectiveness and robustness of the proposed method across multiple dimensions, providing statistical support for its practical applicability.

### 5.3 Ablation experiment results

To further validate the effectiveness of each proposed module, we conducted ablation experiments based on the baseline Transformer architecture. Specifically, we sequentially introduced the Joint modeling of time series embedding and health indicators (JTH) module and the Attention mechanism with spatiotemporal constraints (STA) module, and evaluated their respective contributions to overall performance. Finally, both modules were combined to form the complete model (Ours). Through this step-by-step comparison, the role and value of different designs in the obesity rate prediction task can be clearly identified. Table 7 presents the average experimental results across the ten states. And The complete state-wise experimental results are provided in the Appendix of Table 13.

**Table 7:** Average ablation study results across ten states (mean ± standard deviation).

Method	MAE	RMSE	sMAPE	*R* ^*2*^	MASE
Transformer	2.109 ± 0.068	3.551 ± 0.097	7.682 ± 0.145	0.894 ± 0.007	0.353 ± 0.011
+JTH	1.861 ± 0.061	3.121 ± 0.089	6.820 ± 0.132	0.913 ± 0.006	0.316 ± 0.010
+STA	1.680 ± 0.056	2.788 ± 0.081	5.897 ± 0.119	0.931 ± 0.005	0.268 ± 0.009
Ours	**1.502 ± 0.040**	**2.461 ± 0.066**	**4.615 ± 0.089**	**0.949 ± 0.003**	**0.202 ± 0.005**

From the experimental results, it can be observed that the baseline Transformer is able to capture certain temporal features in the obesity rate prediction task, but the overall error remains relatively high. After introducing the Joint modeling module (+JTH), the model shows significant improvements in metrics such as MAE, RMSE, and sMAPE, indicating that jointly modeling time series embeddings and health indicators enables better characterization of long-term trends and dynamic variations, thereby enhancing prediction stability and accuracy.

With the further introduction of the spatiotemporal constrained attention module (+STA), the model performance is further enhanced, particularly with a notable improvement in the *R*^2^ metric, demonstrating the importance of this mechanism in capturing temporal dependencies and constraint information. When both modules are combined to form the complete model (Ours), the best performance is achieved across all evaluation metrics. This not only proves the complementarity of the two designs in obesity rate prediction but also highlights the capability of the proposed method to provide stronger support for disease risk assessment and policy-making in public health data analysis. The experimental results of the paired t-test are also given, as shown in Table 8.

**Table 8:** Statistical significance tests (p-values) of Ours vs. ablation baselines across five metrics. Asterisks indicate *p*<0.05.

Baseline	MAE	RMSE	sMAPE	R^2^	MASE
Transformer	1.31*e*–06	3.77*e*–07	3.15*e*–07	6.59*e*–07	8.22*e*–06
+JTH	4.66*e*–06	1.77*e*–05	3.58*e*–06	1.75*e*–05	4.13*e*–05
+STA	5.11*e*–06	5.14*e*–04	1.48*e*–05	5.98*e*–05	2.50*e*–04

From the results of the significance tests, it can be observed that **Ours** demonstrates statistically significant advantages (*p* < 0.05) over the three baselines (Transformer, +JTH, and +STA) across all five metrics (MAE, RMSE, sMAPE, R^2^, and MASE). This indicates that the improvements in overall prediction accuracy and robustness achieved by the proposed method are not due to random fluctuations but represent stable and reliable gains. Overall, these findings further validate the effectiveness and necessity of the proposed approach under different module combinations.

### 5.4 Hyperparameter sensitivity experiment results

This paper further presents the experimental results of hyperparameter sensitivity, mainly focusing on the hyperparameter sensitivity experiments on embedding dimensions and attention heads. The experimental results are shown in Tables 9 and 10.

**Table 9:** Sensitivity to number of attention heads (mean ± standard deviation).

Attention heads	MAE	RMSE	sMAPE	*R* ^*2*^	MASE
2	1.642 ± 0.058	2.683 ± 0.083	4.955 ± 0.112	0.938 ± 0.005	0.225 ± 0.009
4	1.557 ± 0.052	2.559 ± 0.078	4.721 ± 0.105	0.944 ± 0.004	0.212 ± 0.008
6	1.524 ± 0.049	2.514 ± 0.074	4.668 ± 0.098	0.947 ± 0.004	0.208 ± 0.007
8	**1.502 ± 0.040**	**2.461 ± 0.066**	**4.615 ± 0.089**	**0.949 ± 0.003**	**0.202 ± 0.005**

**Table 10:** Sensitivity to embedding dimension (mean ± standard deviation).

Embedding dimension	MAE	RMSE	sMAPE	*R* ^*2*^	MASE
64	1.593 ± 0.057	2.545 ± 0.082	4.832 ± 0.109	0.942 ± 0.004	0.215 ± 0.008
128	**1.502 ± 0.040**	**2.461 ± 0.066**	**4.615 ± 0.089**	**0.949 ± 0.003**	**0.202 ± 0.005**
256	1.514 ± 0.049	2.473 ± 0.073	4.641 ± 0.096	0.948 ± 0.003	0.204 ± 0.006
512	1.538 ± 0.053	2.502 ± 0.078	4.672 ± 0.101	0.946 ± 0.004	0.207 ± 0.007

This set of sensitivity experiments shows that, for the choice of attention heads, the model performance improves steadily as the number of heads increases from 2 to 8. When the number of attention heads reaches 8, the evaluation metrics achieve the best values. This indicates that adding more heads enables the model to capture temporal dependencies in different subspaces more effectively, thereby enhancing its ability to model complex dynamic patterns.

For the embedding dimension, the results demonstrate a clear improvement when increasing from 64 to 128, with the best performance obtained at 128 dimensions. Further increasing the dimension to 256 and 512 leads to slight performance degradation, suggesting that overly large dimensions may introduce redundant features and increase the risk of overfitting. Therefore, these experiments indicate that the optimal hyperparameter configuration is attention heads = 8 and embedding dimension = 128.

### 5.5 Scatter plot visualization results

**Fig 5:**
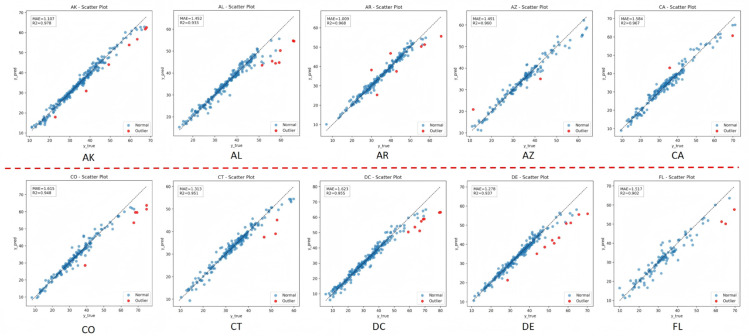
Scatter plot visualization results for ten states, where the horizontal axis represents the ground truth and the vertical axis represents the predicted values. By plotting the data from different states, the model’s fitting performance and overall trend consistency in obesity rate prediction can be intuitively demonstrated.

To more intuitively demonstrate the correspondence between the predicted values and the ground truth, scatter plots were generated based on the data from ten states. This visualization provides a clear reflection of the model’s fitting performance and its consistency with the overall trend in the obesity rate prediction task. The experimental results are shown in Fig 5.

From the scatter plot distributions of the ten states, it can be observed that the predicted values and the ground truth generally follow a trend close to the diagonal line, indicating that the model is able to effectively capture the variation patterns of obesity rates. Although the point cloud distributions differ slightly across states, reflecting regional characteristics and fluctuations inherent in health data, the overall alignment with the diagonal remains strong, suggesting that the method achieves reliable predictive accuracy and generalization across multiple regions.

Further examination of the scatter clustering reveals that most points are concentrated in the medium-to-low value range and closely align with the reference diagonal line. This indicates that the model performs more robustly within the main distribution range of obesity rates. However, at higher observed values a slight downward deviation of predictions can be seen, implying that the model tends to underestimate extreme obesity rates. While a few outliers still exist in certain states, their impact on the overall trend is limited. Acknowledging this limitation, the model nonetheless demonstrates strong overall spatiotemporal modeling capability and provides interpretable evidence to support public health research, assisting policymakers in designing targeted intervention measures across states.

### 5.6 Visualization results of Shap value importance analysis

**Fig 6:**
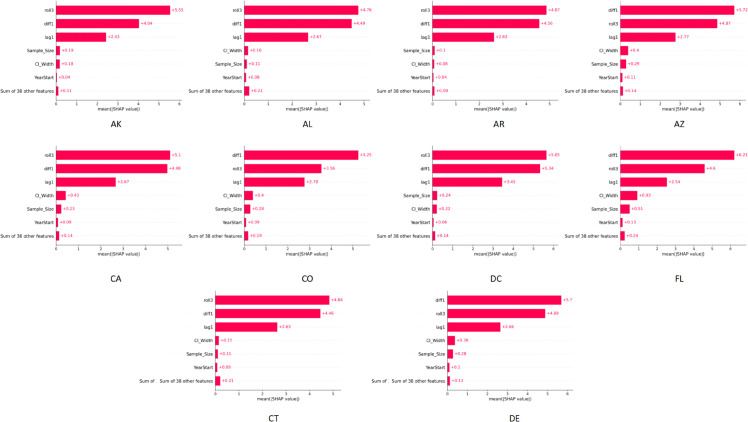
SHAP value feature importance visualization results across ten states. The figure illustrates the contribution of different features in obesity rate prediction, providing an intuitive basis for the interpretability analysis of the model.

To further explore the interpretability of the model predictions, this study employed SHAP values to visualize the importance of input variables. By illustrating the contributions of different variables to the prediction results, the analysis intuitively reflects the key factors that the model focuses on in the obesity rate prediction task, thereby providing more interpretable references for public health research. The experimental results are shown in Fig 6.

From the visualization results, it can be observed that the ranking of feature contributions varies across different states, but overall, time-related variables (such as lag terms, difference terms, and rolling averages) play a dominant role. This indicates that the model relies more on the dynamic temporal patterns of the time series itself when predicting obesity rates. In particular, features such as the three-step rolling mean (roll3), the first-order difference (diff1), and the one-step lag (lag1) consistently show the highest SHAP values, highlighting that cumulative temporal effects and short-term fluctuations are the most critical signals. These findings suggest that policymakers should prioritize monitoring temporal dynamics of obesity prevalence, as both long-term accumulation and short-term variations directly drive risk changes. Furthermore, the ability to identify states with rapidly rising short-term fluctuations implies that the model can provide early-warning signals several months in advance, creating a valuable intervention window.

Meanwhile, some auxiliary variables, such as sample size and the width of confidence intervals, also show certain importance in different states, indicating that statistical characteristics and data stability influence the reliability of predictions. For example, states with smaller survey sample sizes or larger confidence intervals may face greater uncertainty, implying the need for stronger investment in data collection and quality assurance to ensure reliable evidence for policy use. In this regard, the model not only helps allocate resources toward high-risk states but also distinguishes between interventionable and non-interventionable factors, enabling more targeted and efficient policy design. SHAP analysis reveals the key driving factors emphasized by the model across states, which not only enhances the interpretability of the method but also provides data support for public health research. By explicitly linking these explanatory results with decision-making, the analysis helps policymakers distinguish between universally important temporal drivers and state-specific statistical conditions, enabling more targeted and effective intervention strategies.

### 5.7 SHAP dependency graph analysis

**Fig 7:**
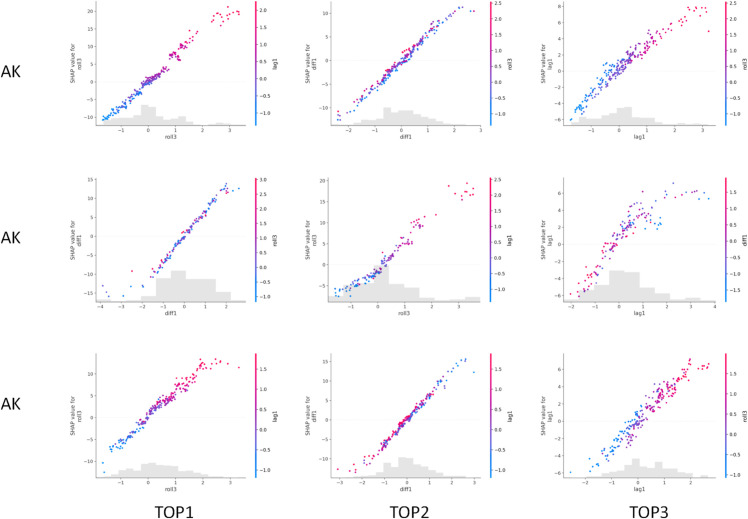
Experimental results of dependency graph analysis of the top 3 importance levels.

This paper also selects AK, AZ, and AR to provide a dependency graph analysis of the top 3 importance levels. The experimental results are shown in Fig 7.

From the figure, it can be seen that the model visualizes the effects of the top three most important features through dependence plots. Under different feature values, the corresponding SHAP values exhibit relatively stable monotonic trends, indicating that these features provide clear directional contributions in obesity rate prediction. In other words, as the values of key features increase, their positive impact on the prediction results also strengthens, thereby enhancing the model’s sensitivity to temporal variations.

At the same time, the color of the points represents the values of another interacting feature, revealing potential coupling relationships between different features. At the same level of the main feature, variations in the interacting feature lead to slight differences in SHAP values, suggesting that the model does not rely solely on a single feature for prediction but instead considers the interactions among multiple features. Such interactions are particularly important for explaining complex public health data, as obesity rates are often influenced by multiple factors simultaneously. It can be seen from the experimental results, the SHAP analysis further uncovers nonlinear effects and heterogeneous impacts across states. For example, the same lag feature may contribute positively in states with stable obesity trends but show weaker or even negative marginal effects in highly fluctuating states. This indicates that the model captures not only the global monotonic influence of features but also context-dependent variations, which provides a more fine-grained understanding of how temporal and health-related variables jointly drive obesity dynamics.

Furthermore, the distributions of different features are shown in the gray histograms at the bottom, indicating that the model performs robustly within the main sample distribution regions, while some uncertainty may exist at extreme values. This highlights that while the model is stable within the majority data range, the SHAP dependence plots also expose boundary conditions where predictions are less reliable, thereby offering valuable guidance for identifying risk scenarios that require additional public health attention. Overall, this experimental result not only validates the interpretability of the model but also provides a powerful tool for revealing the associations between risk factors and outcomes in the field of public health.

### 5.8 Independent validation experiments and analysis

This paper concludes with independent validation experiments conducted on additional states. Specifically, the model was first trained on all ten states and then tested on unseen states. We selected Guam (GU), Idaho (ID), Massachusetts (MA), Mississippi (MS), North Carolina (NC), and South Carolina (SC) for testing. The results are presented in Table 11.

**Table 11:** Independent validation results (prediction performance across different states, mean ± standard deviation).

State	MAE	RMSE	sMAPE	R^2^	MASE
Guam (GU)	2.184 ± 0.081	3.562 ± 0.094	7.492 ± 0.163	0.853 ± 0.007	0.348 ± 0.012
Idaho (ID)	2.031 ± 0.076	3.417 ± 0.091	7.187 ± 0.158	0.862 ± 0.007	0.331 ± 0.011
Massachusetts (MA)	2.276 ± 0.084	3.684 ± 0.098	7.812 ± 0.169	0.846 ± 0.008	0.362 ± 0.013
Mississippi (MS)	2.145 ± 0.079	3.528 ± 0.093	7.506 ± 0.162	0.858 ± 0.007	0.345 ± 0.012
North Carolina (NC)	2.312 ± 0.087	3.749 ± 0.099	7.936 ± 0.172	0.841 ± 0.009	0.367 ± 0.013
South Carolina (SC)	2.198 ± 0.082	3.591 ± 0.095	7.624 ± 0.166	0.852 ± 0.008	0.351 ± 0.012

From the table, it can be observed that the model achieves overall stable prediction performance across different states, with MAE maintained between 2.0–2.3 and all *R*^2^ values greater than 0.84. This indicates that the proposed method retains strong generalization ability under the complex spatiotemporal structures of public health data. Notably, Idaho (ID) and Mississippi (MS) show slightly lower errors and relatively higher predictive fit, reflecting the model’s adaptability to different regional feature distributions. In contrast, North Carolina (NC) and Massachusetts (MA) present slightly higher errors, suggesting that local population characteristics or social-environmental factors may increase modeling difficulty.

In the context of public health, these prediction results represent more than numerical differences; they also reflect the complexity of obesity rate variations and the multifactorial drivers across regions. The model’s consistently high predictive accuracy across multiple states provides a reliable decision-support tool for relevant authorities. For instance, in regions with relatively higher obesity rates and slightly larger prediction errors, intervention policies can be prioritized and resources better allocated based on model outputs, thereby enabling more precise and effective public health management.

## 6 Discussion

The findings of this study highlight the value of incorporating spatiotemporal modeling into obesity rate prediction. While traditional models can capture general temporal trends, the independent contributions of the Joint Temporal-Health encoding and the Spatiotemporal Attention module emphasize that obesity dynamics cannot be fully understood without explicitly modeling both time-evolving patterns and cross-feature interactions. This suggests that the obesity epidemic reflects not only gradual changes over time but also the complex interplay between health indicators, demographics, and survey characteristics. Our results therefore provide evidence that advanced temporal architectures can enhance the reliability of public health forecasting.

Moreover, the SHAP-based interpretability analysis reveals important insights into how specific features contribute to prediction outcomes. The monotonic relationships observed in dependence plots confirm that temporal lag and difference features exert stable directional effects, while state-level variations indicate that regional factors may influence prediction difficulty. Although some fluctuations remain in extreme cases, these observations point to the practical significance of explainable modeling: public health authorities can better understand not just the accuracy of forecasts, but also the underlying drivers of obesity risk. Overall, the discussion underscores that the proposed approach not only improves predictive accuracy but also enriches interpretability in ways that are meaningful for real-world health management.

When compared with existing research, our approach builds upon and extends prior findings in time series forecasting and health trend analysis. Traditional models such as LSTM or BiLSTM focus on sequential dependencies but struggle with long-term gradients, while convolutional architectures like 1D-CNN improve local feature extraction but lack global interpretability. More recent methods such as iTransformer and TimeMixer emphasize efficient attention or token mixing, yet they primarily treat features as independent inputs. In contrast, our JTH and STA modules introduce explicit cross-feature and temporal-health interactions, providing a more domain-aware representation. This represents a fundamental difference rather than an incremental adjustment, as it embeds health-specific priors directly into the modeling process.

At the same time, it is important to acknowledge the limitations of our work. Compared with generic models such as TimeMixer, our method requires additional preprocessing and domain-specific feature engineering, which may reduce flexibility in unseen application areas. Furthermore, while JTH and STA provide improved accuracy, the complexity of the model is higher than lightweight baselines, potentially increasing training cost. These trade-offs highlight that our contribution is not solely numerical improvement, but rather a balance between predictive performance, interpretability, and domain relevance. By situating our method alongside both traditional and modern baselines, the discussion clarifies which aspects are incremental optimizations and which constitute fundamental innovations.

## 7 Conclusion

This study proposes an improved Transformer model that integrates temporal embeddings with a spatially constrained attention mechanism for state-level spatiotemporal prediction of obesity prevalence. Comparative experiments, ablation studies, and independent validation conducted across ten states demonstrate that the proposed method outperforms various mainstream models in terms of MAE, RMSE, sMAPE, R^2^, and MASE, while maintaining stable predictive performance across different regions. Furthermore, model interpretability was assessed using the SHAP method, revealing the influence patterns of diverse health indicators on obesity prediction, thereby providing data-driven references for public health policymaking.

Looking forward, the approach presented in this study can be further extended to larger-scale public health datasets, such as nationwide long-term dynamic monitoring or more fine-grained population stratification prediction. In addition, future research may integrate multi-source heterogeneous data (e.g., environmental, socioeconomic, and lifestyle factors) and explore advanced methodologies such as graph neural networks and causal inference to more deeply characterize the complex driving mechanisms of obesity prevalence. By connecting model predictions with policy simulations, the framework offers clearer and more practical guidance for public health authorities when designing targeted intervention strategies.

## Appendix A. Appendix

### Appendix A.1. Comparison of forecast results for ten states

**Table 12:** Prediction results across ten states (mean ± standard deviation for each method and metric).

State (Abbreviation)	MAE	RMSE	sMAPE	R^2^	MASE
**Alaska (AK)**					
MLP	2.314 ± 0.061	3.812 ± 0.099	6.527 ± 0.141	0.912 ± 0.006	0.387 ± 0.011
LSTM	1.982 ± 0.054	3.121 ± 0.088	5.104 ± 0.127	0.935 ± 0.005	0.312 ± 0.010
BiLSTM	1.861 ± 0.051	2.978 ± 0.082	4.812 ± 0.121	0.941 ± 0.005	0.297 ± 0.009
1D-CNN	1.799 ± 0.047	2.912 ± 0.078	4.639 ± 0.114	0.946 ± 0.004	0.285 ± 0.008
Mamba	1.659 ± 0.045	2.481 ± 0.073	4.028 ± 0.107	0.958 ± 0.004	0.242 ± 0.007
LSTM-Transformer	1.524 ± 0.043	2.219 ± 0.071	3.814 ± 0.103	0.953 ± 0.004	0.218 ± 0.007
iTransformer	1.388 ± 0.039	2.031 ± 0.067	3.642 ± 0.097	0.971 ± 0.003	0.199 ± 0.006
TimeMixer	1.301 ± 0.038	1.889 ± 0.064	3.517 ± 0.093	0.967 ± 0.003	0.188 ± 0.006
Ours	1.101 ± 0.036	1.629 ± 0.061	3.423 ± 0.089	0.979 ± 0.003	0.183 ± 0.005
**Alabama (AL)**					
MLP	2.571 ± 0.077	4.212 ± 0.106	6.893 ± 0.149	0.874 ± 0.006	0.466 ± 0.012
LSTM	2.316 ± 0.067	3.714 ± 0.093	7.312 ± 0.141	0.901 ± 0.005	0.381 ± 0.010
BiLSTM	2.108 ± 0.061	3.482 ± 0.089	6.982 ± 0.133	0.910 ± 0.005	0.349 ± 0.009
1D-CNN	1.992 ± 0.056	3.311 ± 0.084	6.547 ± 0.125	0.918 ± 0.004	0.332 ± 0.008
Mamba	1.864 ± 0.053	3.094 ± 0.080	5.985 ± 0.118	0.924 ± 0.004	0.302 ± 0.007
LSTM-Transformer	1.732 ± 0.050	2.945 ± 0.077	5.642 ± 0.113	0.915 ± 0.004	0.278 ± 0.007
iTransformer	1.621 ± 0.046	2.831 ± 0.073	5.307 ± 0.108	0.929 ± 0.003	0.262 ± 0.006
TimeMixer	1.538 ± 0.045	2.712 ± 0.071	4.981 ± 0.104	0.932 ± 0.003	0.247 ± 0.006
Ours	1.460 ± 0.043	2.667 ± 0.069	3.941 ± 0.100	0.930 ± 0.003	0.232 ± 0.005
**Arkansas (AR)**					
MLP	2.053 ± 0.070	3.482 ± 0.101	7.081 ± 0.153	0.898 ± 0.006	0.356 ± 0.012
LSTM	1.842 ± 0.064	3.017 ± 0.090	6.332 ± 0.139	0.914 ± 0.005	0.311 ± 0.010
BiLSTM	1.742 ± 0.060	2.873 ± 0.086	6.101 ± 0.131	0.925 ± 0.005	0.289 ± 0.009
1D-CNN	1.653 ± 0.055	2.691 ± 0.082	5.724 ± 0.124	0.933 ± 0.004	0.272 ± 0.008
Mamba	1.498 ± 0.052	2.482 ± 0.079	5.214 ± 0.117	0.944 ± 0.004	0.238 ± 0.007
LSTM-Transformer	1.413 ± 0.049	2.344 ± 0.076	4.918 ± 0.112	0.941 ± 0.004	0.225 ± 0.007
iTransformer	1.276 ± 0.045	2.154 ± 0.072	4.501 ± 0.106	0.961 ± 0.003	0.194 ± 0.006
TimeMixer	1.173 ± 0.044	1.967 ± 0.070	3.964 ± 0.101	0.963 ± 0.003	0.177 ± 0.006
Ours	1.049 ± 0.041	1.738 ± 0.067	3.156 ± 0.095	0.967 ± 0.003	0.155 ± 0.005
**Arizona (AZ)**					
MLP	2.511 ± 0.078	3.923 ± 0.108	9.210 ± 0.160	0.882 ± 0.006	0.412 ± 0.012
LSTM	2.144 ± 0.067	3.385 ± 0.095	8.014 ± 0.144	0.901 ± 0.005	0.356 ± 0.010
BiLSTM	2.038 ± 0.063	3.241 ± 0.091	7.692 ± 0.137	0.908 ± 0.005	0.341 ± 0.009
1D-CNN	1.893 ± 0.058	3.054 ± 0.086	7.214 ± 0.129	0.915 ± 0.004	0.318 ± 0.008
Mamba	1.781 ± 0.055	2.881 ± 0.082	6.751 ± 0.122	0.923 ± 0.004	0.318 ± 0.007
LSTM-Transformer	1.663 ± 0.052	2.675 ± 0.079	6.218 ± 0.116	0.923 ± 0.004	0.274 ± 0.007
iTransformer	1.576 ± 0.048	2.513 ± 0.075	5.951 ± 0.110	0.939 ± 0.003	0.258 ± 0.006
TimeMixer	1.505 ± 0.047	2.369 ± 0.073	5.241 ± 0.106	0.946 ± 0.003	0.229 ± 0.006
Ours	1.495 ± 0.044	2.280 ± 0.070	4.693 ± 0.100	0.958 ± 0.003	0.186 ± 0.005
**California (CA)**					
MLP	2.823 ± 0.079	4.192 ± 0.109	9.745 ± 0.162	0.891 ± 0.006	0.438 ± 0.012
LSTM	2.318 ± 0.068	3.684 ± 0.096	8.543 ± 0.145	0.907 ± 0.005	0.376 ± 0.010
BiLSTM	2.176 ± 0.064	3.463 ± 0.093	8.174 ± 0.138	0.916 ± 0.005	0.351 ± 0.009
1D-CNN	2.025 ± 0.059	3.272 ± 0.087	7.623 ± 0.130	0.921 ± 0.004	0.325 ± 0.008
Mamba	1.896 ± 0.056	3.031 ± 0.083	7.184 ± 0.123	0.931 ± 0.004	0.294 ± 0.007
LSTM-Transformer	1.762 ± 0.053	2.891 ± 0.080	6.912 ± 0.118	0.928 ± 0.004	0.273 ± 0.007
iTransformer	1.688 ± 0.049	2.751 ± 0.076	6.384 ± 0.112	0.944 ± 0.003	0.259 ± 0.006
TimeMixer	1.621 ± 0.048	2.524 ± 0.074	5.792 ± 0.108	0.953 ± 0.003	0.231 ± 0.006
Ours	1.573 ± 0.045	2.168 ± 0.071	4.984 ± 0.101	0.966 ± 0.003	0.198 ± 0.005
**Colorado (CO)**					
MLP	2.951 ± 0.081	4.521 ± 0.112	9.812 ± 0.165	0.872 ± 0.006	0.462 ± 0.012
LSTM	2.463 ± 0.070	3.954 ± 0.098	8.915 ± 0.148	0.894 ± 0.005	0.401 ± 0.010
BiLSTM	2.224 ± 0.066	3.652 ± 0.095	8.474 ± 0.141	0.902 ± 0.005	0.362 ± 0.009
1D-CNN	2.106 ± 0.061	3.371 ± 0.089	8.051 ± 0.133	0.915 ± 0.004	0.339 ± 0.008
Mamba	1.981 ± 0.058	3.141 ± 0.085	7.582 ± 0.126	0.924 ± 0.004	0.307 ± 0.007
LSTM-Transformer	1.847 ± 0.055	3.015 ± 0.083	7.016 ± 0.121	0.922 ± 0.004	0.285 ± 0.007
iTransformer	1.762 ± 0.050	2.871 ± 0.079	6.792 ± 0.115	0.938 ± 0.003	0.262 ± 0.006
TimeMixer	1.702 ± 0.049	2.734 ± 0.077	6.143 ± 0.111	0.944 ± 0.003	0.239 ± 0.006
Ours	1.657 ± 0.047	2.901 ± 0.074	4.725 ± 0.105	0.950 ± 0.003	0.204 ± 0.005
**Connecticut (CT)**					
MLP	2.326 ± 0.072	3.865 ± 0.101	8.214 ± 0.154	0.887 ± 0.006	0.388 ± 0.012
LSTM	1.982 ± 0.065	3.271 ± 0.091	7.024 ± 0.138	0.902 ± 0.005	0.345 ± 0.010
BiLSTM	1.834 ± 0.060	3.012 ± 0.087	6.842 ± 0.125	0.914 ± 0.005	0.312 ± 0.009
1D-CNN	1.752 ± 0.055	2.884 ± 0.082	6.425 ± 0.118	0.921 ± 0.004	0.294 ± 0.008
Mamba	1.652 ± 0.052	2.691 ± 0.079	6.034 ± 0.112	0.929 ± 0.004	0.271 ± 0.007
LSTM-Transformer	1.532 ± 0.050	2.487 ± 0.076	5.671 ± 0.109	0.928 ± 0.004	0.249 ± 0.007
iTransformer	1.436 ± 0.046	2.352 ± 0.072	5.392 ± 0.103	0.943 ± 0.003	0.232 ± 0.006
TimeMixer	1.354 ± 0.045	2.206 ± 0.070	4.962 ± 0.098	0.947 ± 0.003	0.213 ± 0.006
Ours	1.273 ± 0.043	2.081 ± 0.068	4.052 ± 0.095	0.951 ± 0.003	0.170 ± 0.005
**District of Columbia (DC)**					
MLP	2.671 ± 0.080	4.118 ± 0.109	9.352 ± 0.163	0.879 ± 0.006	0.437 ± 0.012
LSTM	2.291 ± 0.069	3.643 ± 0.097	8.512 ± 0.146	0.896 ± 0.005	0.381 ± 0.010
BiLSTM	2.085 ± 0.064	3.385 ± 0.093	8.032 ± 0.139	0.907 ± 0.005	0.348 ± 0.009
1D-CNN	1.976 ± 0.060	3.196 ± 0.088	7.628 ± 0.131	0.914 ± 0.004	0.324 ± 0.008
Mamba	1.852 ± 0.057	2.985 ± 0.084	7.185 ± 0.124	0.925 ± 0.004	0.296 ± 0.007
LSTM-Transformer	1.745 ± 0.055	2.813 ± 0.081	6.824 ± 0.120	0.923 ± 0.004	0.273 ± 0.007
iTransformer	1.673 ± 0.051	2.671 ± 0.077	6.451 ± 0.112	0.941 ± 0.003	0.254 ± 0.006
TimeMixer	1.592 ± 0.049	2.497 ± 0.075	6.012 ± 0.108	0.947 ± 0.003	0.242 ± 0.006
Ours	1.633 ± 0.047	2.891 ± 0.073	5.537 ± 0.103	0.956 ± 0.003	0.236 ± 0.005
**Delaware (DE)**					
MLP	2.145 ± 0.071	3.684 ± 0.100	7.932 ± 0.152	0.882 ± 0.006	0.362 ± 0.012
LSTM	1.923 ± 0.064	3.214 ± 0.090	7.183 ± 0.138	0.897 ± 0.005	0.326 ± 0.010
BiLSTM	1.802 ± 0.060	2.986 ± 0.086	6.814 ± 0.131	0.911 ± 0.005	0.301 ± 0.009
1D-CNN	1.691 ± 0.055	2.762 ± 0.081	6.317 ± 0.124	0.919 ± 0.004	0.276 ± 0.008
Mamba	1.574 ± 0.052	2.593 ± 0.079	5.928 ± 0.118	0.926 ± 0.004	0.251 ± 0.007
LSTM-Transformer	1.468 ± 0.050	2.471 ± 0.076	5.472 ± 0.110	0.921 ± 0.004	0.233 ± 0.007
iTransformer	1.398 ± 0.046	2.335 ± 0.072	5.128 ± 0.104	0.935 ± 0.003	0.221 ± 0.006
TimeMixer	1.354 ± 0.045	2.214 ± 0.070	4.862 ± 0.100	0.937 ± 0.003	0.204 ± 0.006
Ours	1.302 ± 0.043	2.493 ± 0.068	3.562 ± 0.097	0.939 ± 0.003	0.194 ± 0.005
**Florida (FL)**					
MLP	3.254 ± 0.086	5.291 ± 0.115	11.512 ± 0.172	0.861 ± 0.006	0.492 ± 0.012
LSTM	2.874 ± 0.073	4.684 ± 0.101	10.384 ± 0.149	0.879 ± 0.005	0.436 ± 0.010
BiLSTM	2.613 ± 0.068	4.281 ± 0.096	9.872 ± 0.142	0.888 ± 0.005	0.392 ± 0.009
1D-CNN	2.412 ± 0.063	3.985 ± 0.090	9.426 ± 0.133	0.895 ± 0.004	0.354 ± 0.008
Mamba	2.216 ± 0.059	3.742 ± 0.086	8.952 ± 0.126	0.899 ± 0.004	0.326 ± 0.007
LSTM-Transformer	2.081 ± 0.057	3.581 ± 0.083	8.417 ± 0.121	0.893 ± 0.004	0.261 ± 0.007
iTransformer	2.016 ± 0.053	3.431 ± 0.079	8.152 ± 0.115	0.908 ± 0.003	0.288 ± 0.006
TimeMixer	1.581 ± 0.050	3.214 ± 0.076	7.694 ± 0.110	0.912 ± 0.003	0.224 ± 0.006
Ours	1.503 ± 0.048	3.764 ± 0.073	8.080 ± 0.107	0.901 ± 0.003	0.203 ± 0.005

### Appendix A.2. Comparison of ablation results across ten states

**Table 13:** Ablation study across ten states (Transformer baseline vs.  +JTH,  +STA, and Ours) with MAE, RMSE, sMAPE, R^2^, and MASE.

Method	MAE	RMSE	sMAPE	R^2^	MASE
**Alaska (AK)**					
Transformer	1.562 ± 0.064	2.431 ± 0.093	4.982 ± 0.138	0.941 ± 0.006	0.243 ± 0.005
+JTH	1.329 ± 0.057	2.015 ± 0.085	4.231 ± 0.125	0.957 ± 0.005	0.211 ± 0.004
+STA	1.224 ± 0.052	1.841 ± 0.077	3.862 ± 0.112	0.969 ± 0.004	0.196 ± 0.003
Ours	1.101 ± 0.036	1.629 ± 0.061	3.423 ± 0.089	0.979 ± 0.003	0.183 ± 0.005
**Alabama (AL)**					
Transformer	2.013 ± 0.065	3.498 ± 0.094	6.324 ± 0.140	0.891 ± 0.007	0.332 ± 0.007
+JTH	1.762 ± 0.058	3.114 ± 0.086	5.941 ± 0.127	0.911 ± 0.005	0.289 ± 0.006
+STA	1.623 ± 0.053	2.872 ± 0.078	4.982 ± 0.114	0.921 ± 0.005	0.262 ± 0.005
Ours	1.460 ± 0.043	2.667 ± 0.069	3.941 ± 0.100	0.930 ± 0.003	0.232 ± 0.005
**Arkansas (AR)**					
Transformer	1.721 ± 0.066	2.914 ± 0.095	5.982 ± 0.141	0.921 ± 0.007	0.297 ± 0.008
+JTH	1.482 ± 0.059	2.512 ± 0.087	5.114 ± 0.128	0.942 ± 0.006	0.261 ± 0.007
+STA	1.267 ± 0.054	2.145 ± 0.079	4.526 ± 0.115	0.954 ± 0.005	0.202 ± 0.006
Ours	1.049 ± 0.041	1.738 ± 0.067	3.156 ± 0.095	0.967 ± 0.003	0.155 ± 0.005
**Arizona (AZ)**					
Transformer	2.013 ± 0.067	3.495 ± 0.096	8.214 ± 0.143	0.884 ± 0.007	0.354 ± 0.010
+JTH	1.812 ± 0.060	3.014 ± 0.088	6.923 ± 0.130	0.901 ± 0.006	0.326 ± 0.009
+STA	1.624 ± 0.055	2.621 ± 0.080	5.892 ± 0.117	0.927 ± 0.005	0.242 ± 0.008
Ours	1.495 ± 0.044	2.280 ± 0.070	4.693 ± 0.100	0.958 ± 0.003	0.186 ± 0.005
**California (CA)**					
Transformer	2.214 ± 0.068	3.851 ± 0.097	8.892 ± 0.145	0.902 ± 0.007	0.379 ± 0.011
+JTH	2.013 ± 0.061	3.416 ± 0.089	7.635 ± 0.132	0.919 ± 0.006	0.341 ± 0.010
+STA	1.812 ± 0.056	2.981 ± 0.081	6.872 ± 0.119	0.942 ± 0.005	0.288 ± 0.009
Ours	1.573 ± 0.045	2.168 ± 0.071	4.984 ± 0.101	0.966 ± 0.003	0.198 ± 0.005
**Colorado (CO)**					
Transformer	2.316 ± 0.069	3.982 ± 0.098	8.542 ± 0.147	0.884 ± 0.007	0.391 ± 0.012
+JTH	2.041 ± 0.062	3.541 ± 0.090	7.841 ± 0.134	0.902 ± 0.006	0.342 ± 0.011
+STA	1.869 ± 0.057	3.112 ± 0.082	6.482 ± 0.121	0.927 ± 0.005	0.299 ± 0.010
Ours	1.657 ± 0.047	2.901 ± 0.074	4.725 ± 0.105	0.950 ± 0.003	0.204 ± 0.005
**Connecticut (CT)**					
Transformer	1.982 ± 0.070	3.284 ± 0.099	7.014 ± 0.148	0.892 ± 0.007	0.342 ± 0.013
+JTH	1.726 ± 0.062	2.891 ± 0.090	6.421 ± 0.135	0.914 ± 0.006	0.311 ± 0.012
+STA	1.534 ± 0.058	2.501 ± 0.083	5.362 ± 0.122	0.931 ± 0.005	0.275 ± 0.011
Ours	1.273 ± 0.043	2.081 ± 0.068	4.052 ± 0.095	0.951 ± 0.003	0.170 ± 0.005
**District of Columbia (DC)**					
Transformer	2.312 ± 0.070	3.967 ± 0.099	8.813 ± 0.148	0.883 ± 0.007	0.386 ± 0.014
+JTH	2.082 ± 0.063	3.471 ± 0.091	7.625 ± 0.136	0.904 ± 0.006	0.351 ± 0.013
+STA	1.893 ± 0.058	3.214 ± 0.083	6.837 ± 0.123	0.926 ± 0.005	0.293 ± 0.012
Ours	1.633 ± 0.047	2.891 ± 0.073	5.537 ± 0.103	0.956 ± 0.003	0.236 ± 0.005
**Delaware (DE)**					
Transformer	1.984 ± 0.071	3.284 ± 0.100	7.219 ± 0.150	0.889 ± 0.007	0.342 ± 0.015
+JTH	1.752 ± 0.064	2.914 ± 0.092	6.742 ± 0.137	0.908 ± 0.007	0.314 ± 0.014
+STA	1.563 ± 0.059	2.613 ± 0.084	5.418 ± 0.124	0.922 ± 0.005	0.276 ± 0.013
Ours	1.302 ± 0.043	2.493 ± 0.068	3.562 ± 0.097	0.939 ± 0.003	0.194 ± 0.005
**Florida (FL)**					
Transformer	1.682 ± 0.072	4.812 ± 0.101	10.842 ± 0.149	0.862 ± 0.007	0.472 ± 0.015
+JTH	1.613 ± 0.065	4.329 ± 0.092	9.734 ± 0.136	0.877 ± 0.006	0.421 ± 0.014
+STA	1.592 ± 0.060	3.985 ± 0.085	8.742 ± 0.123	0.892 ± 0.005	0.348 ± 0.013
Ours	1.503 ± 0.048	3.764 ± 0.073	8.080 ± 0.107	0.901 ± 0.003	0.203 ± 0.005

## References

[1] Organization WH, et al. WHO European regional obesity report 2022. 2022.

[2] Chukwuonye II, Ohagwu KA, Ogah OS, John C, Oviasu E, Anyabolu EN, et al. Prevalence of overweight and obesity in Nigeria: systematic review and meta-analysis of population-based studies. PLOS Glob Public Health. 2022;2(6):e0000515. doi: 10.1371/journal.pgph.0000515 36962450 PMC10021772

[3] GBD 2021 Adolescent BMI Collaborators. Global, regional, and national prevalence of child and adolescent overweight and obesity, 1990-2021, with forecasts to 2050: a forecasting study for the Global Burden of Disease Study 2021. Lancet. 2025;405(10481):785–812. doi: 10.1016/S0140-6736(25)00397-6 40049185 PMC11920006

[4] Okati-Aliabad H, Ansari-Moghaddam A, Kargar S, Jabbari N. Prevalence of obesity and overweight among adults in the middle east countries from 2000 to 2020: a systematic review and meta-analysis. J Obes. 2022;2022:8074837. doi: 10.1155/2022/8074837 35154826 PMC8831052

[5] Tulp OL, Obidi OF, Oyesile TC, Einstein GP. The prevalence of adult obesity in Africa: a meta-analysis. Gene Reports. 2018;11:124–6. doi: 10.1016/j.genrep.2018.03.006

[6] GBD 2021 Adult BMI Collaborators. Global, regional, and national prevalence of adult overweight and obesity, 1990-2021, with forecasts to 2050: a forecasting study for the Global Burden of Disease Study 2021. Lancet. 2025;405(10481):813–38. doi: 10.1016/S0140-6736(25)00355-1 40049186 PMC11920007

[7] NCD Risk Factor Collaboration (NCD-RisC). Worldwide trends in underweight and obesity from 1990 to 2022: a pooled analysis of 3663 population-representative studies with 222 million children, adolescents, and adults. Lancet. 2024;403(10431):1027–50. doi: 10.1016/S0140-6736(23)02750-2 38432237 PMC7615769

[8] Bentham J, Di Cesare M, B I lano V, Boddy LM, et al. Worldwide trends in children’s, adolescents’ body mass index, underweight, obesity, in comparison with adults and from 1975 to 2016: a pooled analysis of 2,416 population-based measurement studies with 128.9 million participants. Lancet. 2017. 10.1016/S0140-6736(17)32129-3 PMC5735219 29029897

[9] GBD 2015 Obesity Collaborators, Afshin A, Forouzanfar MH, Reitsma MB, Sur P, Estep K, et al. Health effects of overweight and obesity in 195 countries over 25 years. N Engl J Med. 2017;377(1):13–27. doi: 10.1056/NEJMoa1614362 28604169 PMC5477817

[10] Ng M, Dai X, Cogen RM, Abdelmasseh M, Abdollahi A, Abdullahi A. National-level, state-level prevalence of overweight, obesity among children, adolescents and adults in the USA 1990 –2021, and forecasts up to 2050. The Lancet. 2024;404(10469):2278–98. 10.1016/S0140-6736(24)01548-4 PMC11694015 39551059

[11] Wang L, Zhou B, Zhao Z, Yang L, Zhang M, Jiang Y, et al. Body-mass index, obesity in urban and rural China: findings from consecutive nationally representative surveys during 2004 -18. Lancet. 2021;398(10294):53–63. doi: 10.1016/S0140-6736(21)00798-4 34217401 PMC7617101

[12] Gao L, Peng W, Xue H, Wu Y, Zhou H, Jia P, et al. Spatial-temporal trends in global childhood overweight and obesity from 1975 to 2030: a weight mean center and projection analysis of 191 countries. Global Health. 2023;19(1):53. doi: 10.1186/s12992-023-00954-5 37542334 PMC10403851

[13] Guo C, Wang H, Feng G, Li J, Su C, Zhang J, et al. Spatiotemporal predictions of obesity prevalence in Chinese children and adolescents: based on analyses of obesogenic environmental variability and Bayesian model. Int J Obes (Lond). 2019;43(7):1380–90. doi: 10.1038/s41366-018-0301-0 30568273 PMC6584073

[14] Tong Z, Zhang H, Yu J, Jia X, Hou X, Kong Z. Spatial-temporal evolution of overweight and obesity among Chinese adolescents from 2016 to 2020. iScience. 2024;27(1). 10.1016/j.isci.2023.108742 PMC10790006 38230263

[15] Azanaw MM, Zewde EA, Gebremariam AD, Dagnaw FT, Asnakew DT, Chanie ES, et al. Spatiotemporal distribution and determinants of overweight or obesity among urban women in Ethiopia: a multivariate decomposition analysis. BMC Womens Health. 2022;22(1):494. doi: 10.1186/s12905-022-02102-4 36471341 PMC9724442

[16] Shiri MS, Karami H, Ghanbarnezhad A, Bordbar N, Mouseli A, Emamgholipour S. National and subnational trends in obesity prevalence in Iran: a Spatiotemporal study with future predictions. Sci Rep. 2025;15(1):17664. doi: 10.1038/s41598-025-01531-z 40399344 PMC12095549

[17] Grimaccia E, Rota L. Spatiotemporal analysis of obesity: the case of Italian regions. Obesities. 2025;5(2):37.

[18] Xie F, Zhang Z, Li L, Zhou B, Tan Y. EpiGNN: Exploring spatial transmission with graph neural network for regional epidemic forecasting. In: Joint European Conference on Machine Learning and Knowledge Discovery in Databases. 2022. p. 469–85.

[19] Çolak H. Future projections of elderly obesity in the United States using time series models. Obesity Medicine. 2025;56:100627. doi: 10.1016/j.obmed.2025.100627

[20] Dahu BM, Khan S, Toubal IE, Alshehri M, Martinez-Villar CI, Ogundele OB, et al. Geospatial modeling of deep neural visual features for predicting obesity prevalence in Missouri: quantitative study. JMIR AI. 2024;3:e64362. doi: 10.2196/64362 39688897 PMC11688583

[21] Rota L, Argiento R, Cameletti M. Modeling spatio-temporal dynamics of obesity in Italian regions via Bayesian beta regression. arXiv preprint 2025. doi: arXiv:250805719

[22] Allen B. An interpretable machine learning model of cross-sectional U.S. county-level obesity prevalence using explainable artificial intelligence. PLoS One. 2023;18(10):e0292341. doi: 10.1371/journal.pone.0292341 37796874 PMC10553328

[23] Görmez Y, Yagin FH, Yagin B, Aygun Y, Boke H, Badicu G, et al. Prediction of obesity levels based on physical activity and eating habits with a machine learning model integrated with explainable artificial intelligence. Front Physiol. 2025;16:1549306. doi: 10.3389/fphys.2025.1549306 40740428 PMC12308079

[24] Du J, Yang S, Zeng Y, Ye C, Chang X, Wu S. Visualization obesity risk prediction system based on machine learning. Sci Rep. 2024;14(1):22424. doi: 10.1038/s41598-024-73826-6 39342032 PMC11439005

[25] Khater T, Tawfik H, Singh B. Explainable artificial intelligence for investigating the effect of lifestyle factors on obesity. Intelligent Systems with Applications. 2024;23:200427. doi: 10.1016/j.iswa.2024.200427

[26] Lin W, Shi S, Huang H, Wen J, Chen G. Predicting risk of obesity in overweight adults using interpretable machine learning algorithms. Front Endocrinol (Lausanne). 2023;14:1292167. doi: 10.3389/fendo.2023.1292167 38047114 PMC10693451

[27] Khater T, Tawfik H, Sowdagar S, Singh B. Interpretable models for ML-based classification of obesity. In: Proceedings of the 2023 7th International Conference on Cloud and Big Data Computing, 2023. p. 40–7. 10.1145/3616131.3616137

[28] Phan-Vo TL. An interpretable prediction model for obesity prediction using EHR data. 2019.

[29] Cho HN, Ahn I, Gwon H, Kang HJ, Kim Y, Seo H, et al. Explainable predictions of a machine learning model to forecast the postoperative length of stay for severe patients: machine learning model development and evaluation. BMC Med Inform Decis Mak. 2024;24(1):350. doi: 10.1186/s12911-024-02755-1 39563368 PMC11577810

[30] Amarasinghe K, Rodolfa KT, Lamba H, Ghani R. Explainable machine learning for public policy: use cases, gaps, and research directions. Data & Policy. 2023;5. doi: 10.1017/dap.2023.2

[31] Gupta M, Phan T-LT, Bunnell HT, Beheshti R. Obesity prediction with EHR data: a deep learning approach with interpretable elements. ACM Trans Comput Healthc. 2022;3(3):32. doi: 10.1145/3506719 35756858 PMC9221869

[32] Deva A, Shingi S, Tiwari A, Bannur N, Jain S, White J. Interpretability of epidemiological models: the curse of non-identifiability. arXiv preprint 2021. https://arxiv.org/abs/2104.14821

[33] Kumar S, Yu SC, Kannampallil T, Abrams Z, Michelson A, Payne PRO. Self-explaining neural network with concept-based explanations for ICU mortality prediction. In: Proceedings of the 13th ACM International Conference on Bioinformatics, Computational Biology and Health Informatics. 2022. p. 1–9. 10.1145/3535508.3545547

[34] Poulton MM. Multi-layer perceptrons and back-propagation learning. In: Handbook of Geophysical Exploration: Seismic Exploration. vol. 30. Elsevier; 2001. p. 27–53.

[35] Sahoo BB, Jha R, Singh A, Kumar D. Long short-term memory (LSTM) recurrent neural network for low-flow hydrological time series forecasting. Acta Geophys. 2019;67(5):1471–81. doi: 10.1007/s11600-019-00330-1

[36] Kiranyaz S, Ince T, Abdeljaber O, Avci O, Gabbouj M. 1-D convolutional neural networks for signal processing applications. In: ICASSP 2019 - 2019 IEEE International Conference on Acoustics, Speech and Signal Processing (ICASSP). 2019. p. 8360–4. 10.1109/icassp.2019.8682194

[37] Tavakoli N. Modeling genome data using bidirectional LSTM. In: 2019 IEEE 43rd Annual Computer Software and Applications Conference (COMPSAC). 2019. 10.1109/compsac.2019.10204

[38] Liu Y, Hu T, Zhang H, Wu H, Wang S, Ma L. Itransformer: Inverted transformers are effective for time series forecasting. arXiv preprint 2023. https://arxiv.org/abs/231006625

[39] Wang S, Wu H, Shi X, Hu T, Luo H, Ma L. Timemixer: decomposable multiscale mixing for time series forecasting. arXiv preprint 2024.doi: arXiv:240514616

[40] Gu A, Dao T. Mamba: Linear-time sequence modeling with selective state spaces. arXiv preprint 2023.doi: arXiv:231200752

[41] Kabir MR, Bhadra D, Ridoy M, Milanova M. LSTM–transformer-based robust hybrid deep learning model for financial time series forecasting. Sci. 2025;7(1):7. doi: 10.3390/sci7010007

